# Clinical applications of gene therapy for rare diseases: A review

**DOI:** 10.1111/iep.12478

**Published:** 2023-05-13

**Authors:** Ioannis Papaioannou, James S. Owen, Rafael J. Yáñez‐Muñoz

**Affiliations:** ^1^ Division of Medicine University College London London UK; ^2^ AGCTlab.org Centre of Gene and Cell Therapy Centre for Biomedical Sciences Department of Biological Sciences School of Life Sciences and the Environment Royal Holloway University of London Egham UK

**Keywords:** adeno‐associated virus vectors, antisense oligonucleotides, CAR‐T cells, retrovirus vectors

## Abstract

Rare diseases collectively exact a high toll on society due to their sheer number and overall prevalence. Their heterogeneity, diversity, and nature pose daunting clinical challenges for both management and treatment. In this review, we discuss recent advances in clinical applications of gene therapy for rare diseases, focusing on a variety of viral and non‐viral strategies. The use of adeno‐associated virus (AAV) vectors is discussed in the context of Luxturna, licenced for the treatment of *RPE65* deficiency in the retinal epithelium. Imlygic, a herpes virus vector licenced for the treatment of refractory metastatic melanoma, will be an example of oncolytic vectors developed against rare cancers. Yescarta and Kymriah will showcase the use of retrovirus and lentivirus vectors in the autologous ex vivo production of chimeric antigen receptor T cells (CAR‐T), licenced for the treatment of refractory leukaemias and lymphomas. Similar retroviral and lentiviral technology can be applied to autologous haematopoietic stem cells, exemplified by Strimvelis and Zynteglo, licenced treatments for adenosine deaminase‐severe combined immunodeficiency (ADA‐SCID) and β‐thalassaemia respectively. Antisense oligonucleotide technologies will be highlighted through Onpattro and Tegsedi, RNA interference drugs licenced for familial transthyretin (TTR) amyloidosis, and Spinraza, a splice‐switching treatment for spinal muscular atrophy (SMA). An initial comparison of the effectiveness of AAV and oligonucleotide therapies in SMA is possible with Zolgensma, an AAV serotype 9 vector, and Spinraza. Through these examples of marketed gene therapies and gene cell therapies, we will discuss the expanding applications of such novel technologies to previously intractable rare diseases.

## 1 INTRODUCTION

This review outlines current gene therapy strategies to treat rare diseases (RDs). In‐depth analysis or a full overview of the RD field is beyond our scope, but other reviews are available.^1^, ^2^, ^3^ Though definitions for RDs vary, the defining factor is low prevalence, typically <0.05%.^1^ There are nearly 10,000 RDs that cumulatively affect over 5% of the global population, about 400 million people, thus exacting a high global health burden.^4^ Their phenotype spectrum is extremely diverse, ranging from mild, for example, Inherited Macroglossia,^5^, ^6^ to severe, for example, Huntington's chorea^7^, ^8^ or adenosine deaminase—severe combined immunodeficiency (ADA‐SCID).^9^, ^10^ Approx. 80% of RDs involve genetic alterations,^11^ and typically for each disease, there exist multiple different causative mutations with important implications for disease management. RDs also include some infectious diseases, such as Babesiosis,^12^, ^13^ a tick‐borne infection.

The healthcare cost for RDs is high; they can be chronic, often have devastating consequences and effective treatments are lacking, typically translating into extensive and expensive symptomatic management, including hospitalization. Undiagnosed RDs compound the problem. Without underlying cause identification, managing patient symptoms is inefficient and ineffective, worsening outcomes and increasing healthcare resource consumption.^14^, ^15^, ^16^ Cumulatively, in developed countries RDs account for ~10% of total direct healthcare spending for a patient population of 5%–7%.^17^, ^18^

Research and development for disease treatments are expensive and protracted, regardless of patient numbers. For common diseases with a patient base of millions, they deliver value and can be funded largely by the patients themselves directly or indirectly. This is not the case for RDs whose patient base typically ranges from a few thousands to a few hundred thousand but can be as low as a single patient. Treatment development for RDs may not be commercially viable, but the suffering and high healthcare costs imposed by RDs, make it worthwhile for governments to step in. Initiatives such as the orphan drug designation status^19^, ^20^, ^21^ have been instrumental in incentivizing pharmaceutical companies and spurring innovation.

As a group, the nature of RDs largely precludes small molecule therapeutics; functions of aberrant or missing genes are not readily replaced by other molecules. Successes, such as Imatinib for acute lymphoblastic leukaemia (ALL) are exceptions.^22^ Biologic therapeutics such as protein supplementation can offer solutions but often fail to fully restore homeostatic balance, offering only partial symptom relief. Moreover, the development of one biologic agent benefits only modestly from work done on previous agents and their applicability is not universal. Haemophilia A,^23^, ^24^ affects just one protein and is effectively treated with recombinant Factor VIII. This is not the case for 47XXY (occasionally also 48XXYY) Klinefelter syndrome,^25^, ^26^ a congenital X and Y chromosome duplication, which profoundly impacts global gene expression patterns. Its correction is beyond current technological capabilities leaving symptomatic management as the only option.

By contrast, nucleic‐acid‐based therapies are exceptionally well‐suited to treat RDs, because (i) the nucleic‐acid payload is interchangeable, so platform and delivery developments can benefit many different disease areas; and (ii) the internal homeostatic balance is more effectively restored, either permanently or transiently depending on the technology used, to confer greater protection against the disease‐inflicted damage. In brief, gene therapy promises more effective treatments and a much more efficient therapeutic discovery process.

Here, we discuss current clinical development and practice of gene therapy for RDs. We focus almost exclusively on treatments that have received regulatory approval and are being used in people affected, contrasting them to conventional therapeutics and illustrating the wider applicability of their platforms.

**FIGURE 1:**
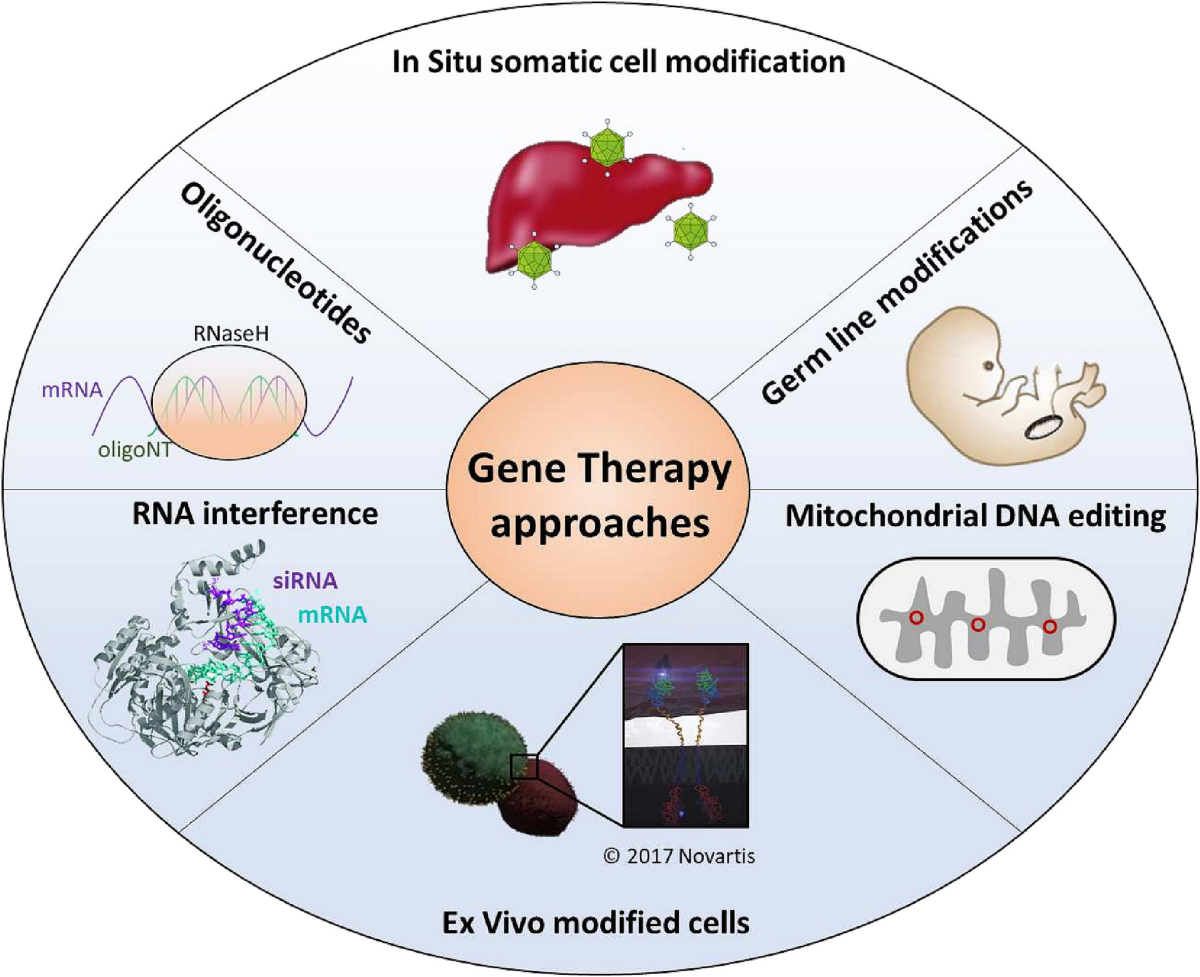
The expanding Gene Therapy field. Originally gene therapy was envisioned as the in situ modification of genetic information of cells within tissues. The field has evolved beyond that encompassing more aspects of nucleic acid technology, particularly oligonucleotide technology, which aims to modify gene expression, without necessarily changing the cell's genetic information. The modification of a patient's cells ex vivo, outside the body prior to reimplantation has proven to be a successful clinical strategy. Although recent technological advancements have now enabled mitochondrial and germ line or embryonic cell gene therapy, these approaches are not yet being used due to safety and ethical issues.

Originally envisaged as alleviating or curing disease by correcting defective genes, gene therapy has evolved to encompass several therapeutic interventions (Figure 1). Genetic defects can cause disease by abolishing, reducing or increasing the expression of one or more proteins, or by creating novel proteins with altered functions (gain‐of‐function). The scale of these defects varies widely from point mutations to multi‐nucleotide deletions or insertions, gene copy number variation and karyotypic alterations. Current technology limits gene therapy to individual gene defects, but recent advances have the potential to correct larger scale abnormalities. To facilitate our study of the subject, we will divide our discussion of successful clinical applications of gene therapy into the following broad categories:

1. Direct modification of somatic cell DNA in vivo.
2. Modification of DNA in differentiated somatic cells, prior to reimplantation.
3. Modification of DNA in stem cells, prior to reimplantation.
4. Manipulation of post‐transcriptional RNA processing and translation with nucleic acid technology.

## 2 DIRECT MODIFICATION OF SOMATIC CELL DNA IN VIVO

### 2.1 Gene supplementation in somatic and post‐mitotic tissues: Luxturna AAV‐based gene supplementation treatment for LCA2

**FIGURE 2:**
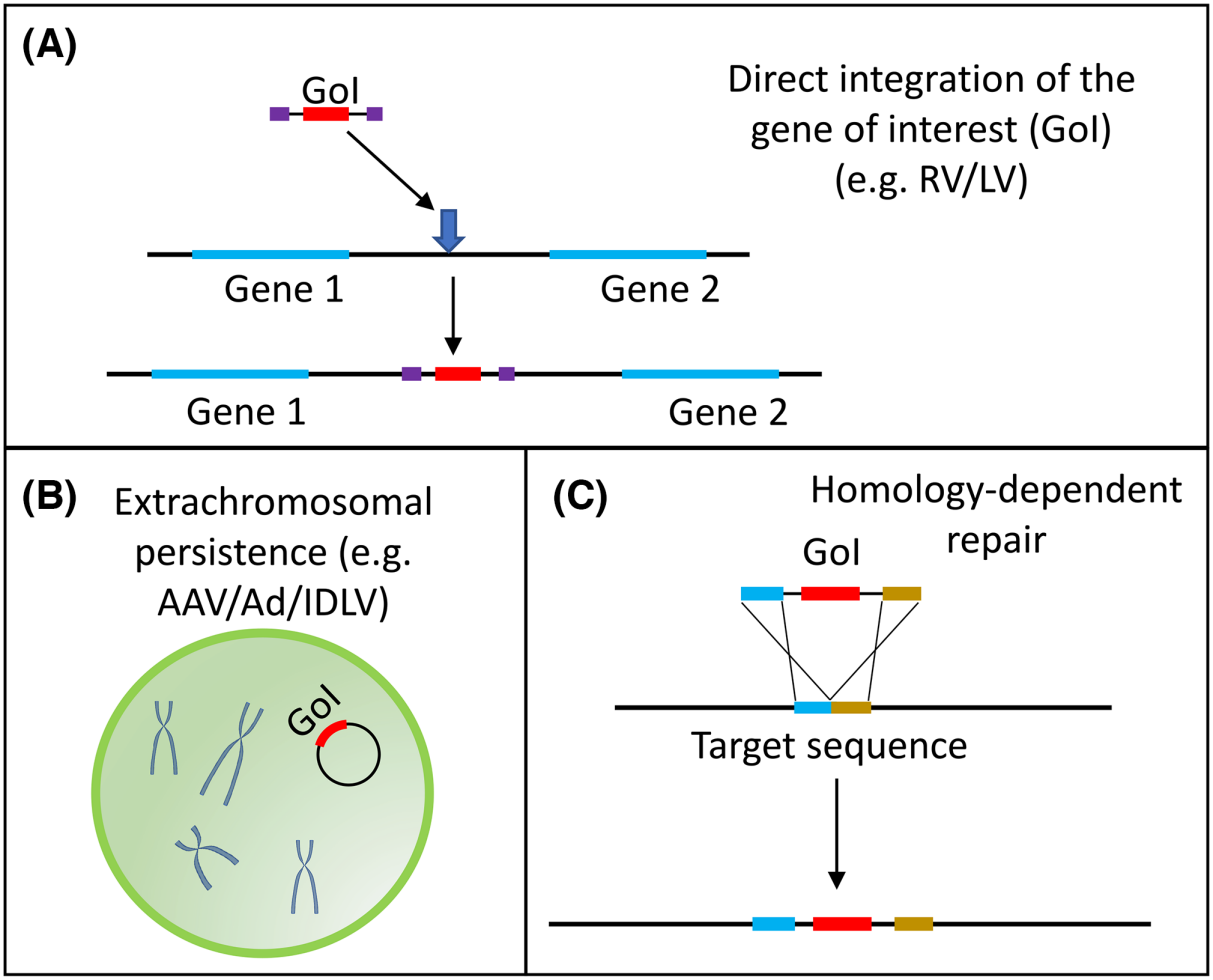
Common gene supplementation strategies. (A) A gene of interest (GoI) can be incorporated into a chromosomal break, which may disrupt an existing gene, as the insertion point may be random. Examples include integrating viral vectors (RV, retrovirus vector; LV, lentivirus vector) and transposons. (B) Persistence of the new genetic material as an extrachromosomal element. Adeno‐associated virus (AAV), adenovirus (Ad) and integration‐deficient lentivirus vectors (IDLVs) are common examples. Without matrix‐attachment region and sequences directing replication, the extrachromosomal element will be diluted out through cell division. (C) Homology‐dependent repair (HDR) involves the targeted replacement of a host sequence. It is the safest method, yet also the hardest to harness.

The absence of a functional copy of a gene, key to the function of a highly differentiated tissue (e.g. lung or eye) is a common cause of RDs. Such cases lend themselves to direct addition of a functional gene copy to cells of the target tissue. This is gene supplementation: delivery of DNA containing the gene of interest to the nucleus, while ensuring its expression and persistence therein (Figure 2). Viruses can be engineered into powerful gene supplementation platforms. The basic premise is to create a custom viral genome with the gene of interest replacing viral genes and artificially package it into virions. These virions can transduce cells and deliver the target gene but cannot replicate or cause disease. As an example, we shall look at the recent successful clinical use of viral vector technology in inherited retinal dystrophies.

**FIGURE 3:**
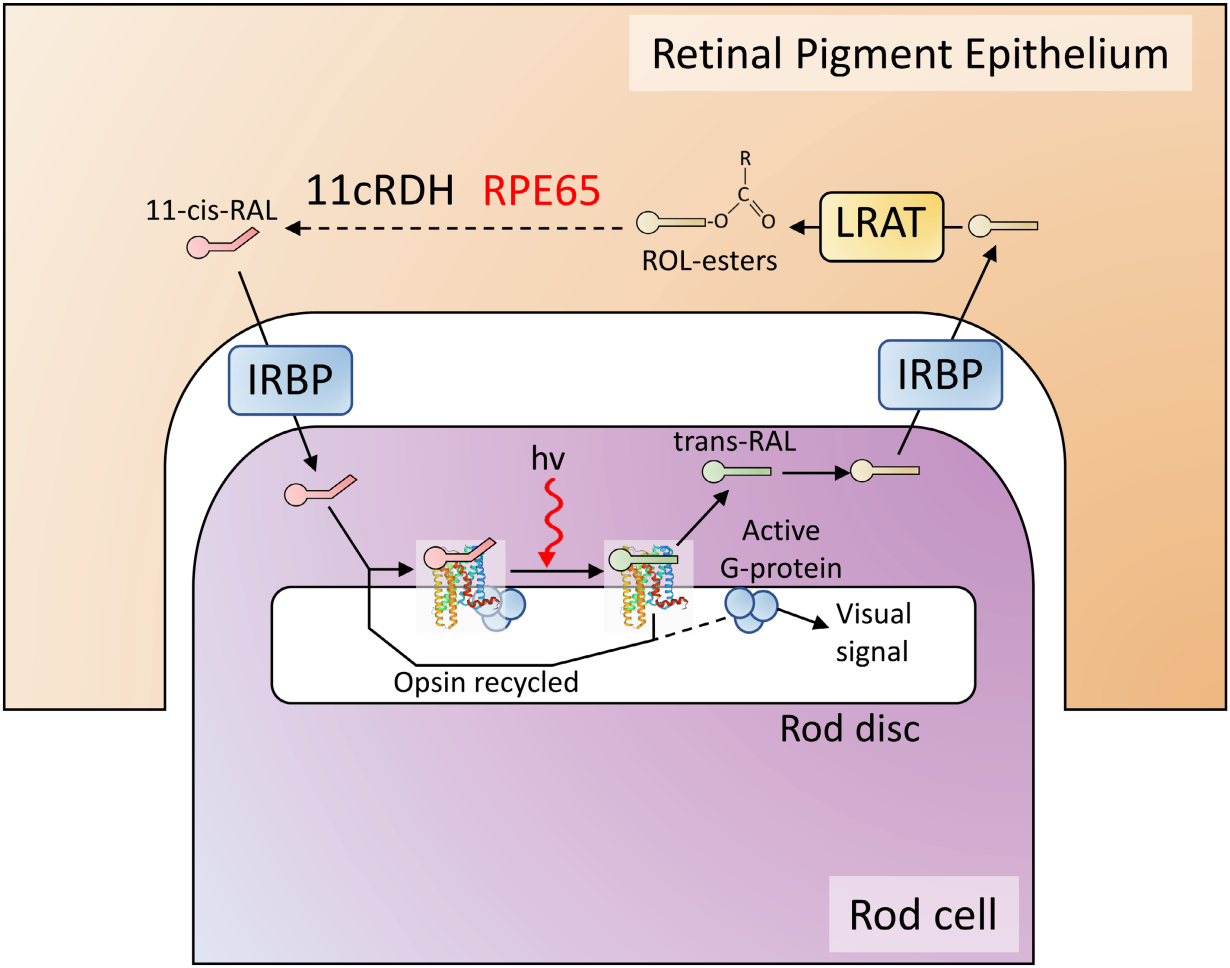
The visual cycle in rod cells. The optical signal is generated by the opsin proteins with the help of 11‐cis retinal (RAL), which absorbs light, changes to all‐trans retinal and in the process activates opsin. All‐trans retinal is no longer photosensitive and needs to be converted back to 11‐cis RAL. This conversion is not carried out by the rod cells themselves but by the supporting retinal pigment epithelium (RPE). Trans‐RAL is released from opsin and since it is membrane permeable it is transported with the help of special carrier proteins (interphotoreceptor retinoid‐binding protein, IRBP) in the extracellular matrix to the RPE, where a series of specialized enzymes catalyse the conversion. The 11‐cis‐retinal product is transported back to the rod cells. Metabolic defects in the RPE enzymes block the conversion of 11‐cis retinal and lead to accumulation of various intermediates such as retinol (ROL) esters, which can reach toxic levels. External supply of 11‐cis retinal and removal of the intermediates via the blood is not sufficient to maintain vision.

The retinal pigment epithelium‐specific 65‐kDa protein (RPE65) is an enzyme critical for the regeneration of 11‐cis‐retinal during the visual cycle^27^, ^28^ (Figure 3). Without RPE65, 11‐cis‐retinal is depleted, leaving the photoreceptors unable to operate, while other intermediates in the metabolic pathway build up to potentially toxic levels. *RPE65* mutations cause a spectrum of inherited retinal dystrophies, which result in blindness at birth or very early childhood.^27^, ^28^ The most common phenotypes are Leber's Congenital Amaurosis^29^, ^30^ and Retinitis Pigmentosa,^31^ but other rarer phenotypes are also possible depending on the *RPE65* genetic defect.^28^

Replacement of RPE65 function in the patient's eye cannot currently be achieved by means other than gene therapy and is an attractive gene therapy target (see Figure 3). An intense research and development effort culminated in the development of Voretigene neparvovec, an adeno‐associated virus (AAV) RPE65 gene replacement platform.^32^, ^33^, ^34^, ^35^, ^36^, ^37^ In 2017, it was approved by the US Food and Drug Administration (FDA), under the commercial name Luxturna for the treatment of type 2 Leber's Congenital Amaurosis (LCA2).^38^

**FIGURE 4:**
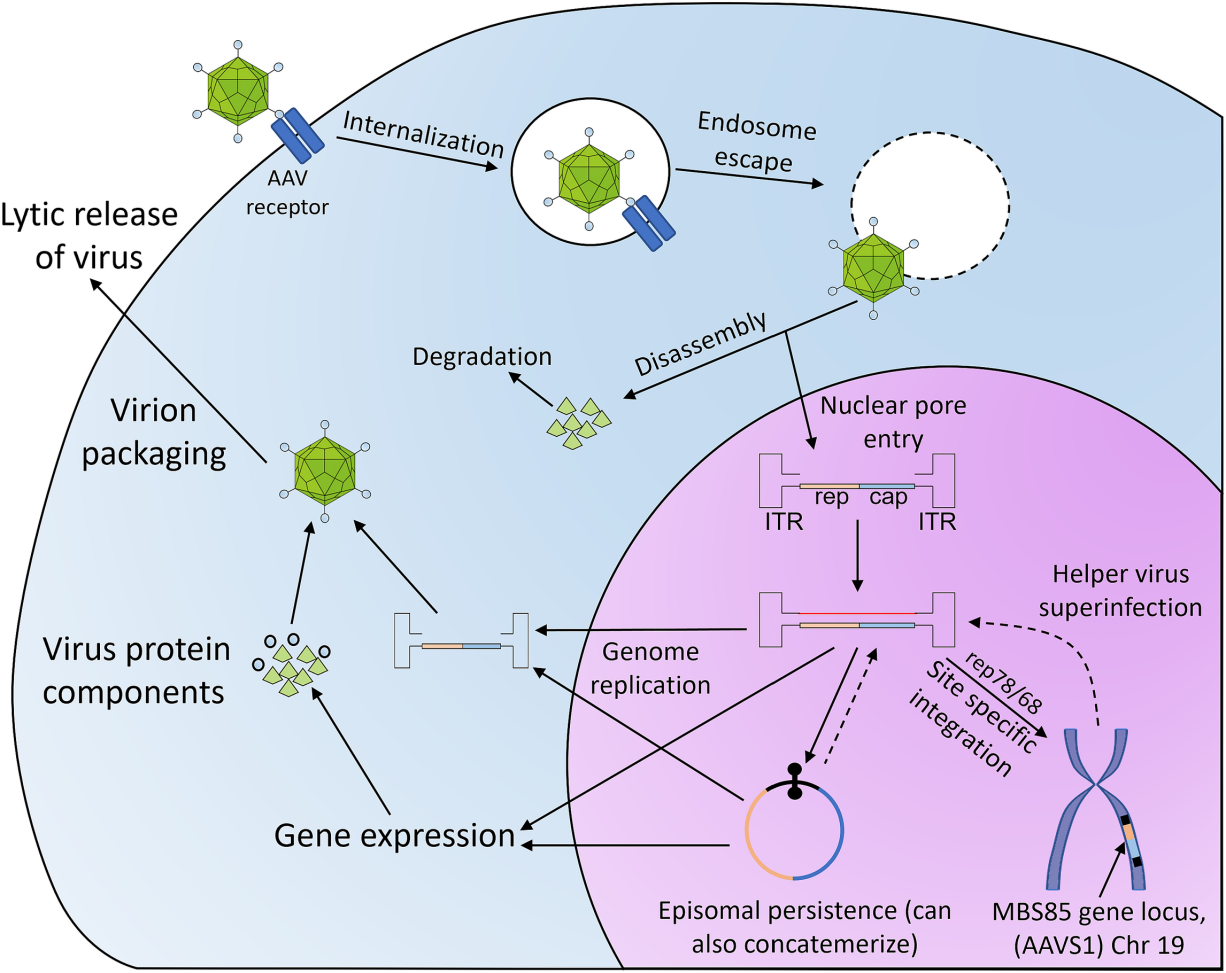
The lifecycle of adeno‐associated virus (AAV). AAV enters the cells via receptor‐mediated endocytosis and then disrupts the endosome to escape into the cytoplasm. The capsid disassembles and simultaneously passes the viral genome onto nuclear pores facilitating nuclear entry. In the nucleus, the AAV genome, which is single‐stranded, makes use of the inverted terminal repeats (ITRs) to become double‐stranded. In the absence of a concomitant helper virus, AAV goes dormant, preferentially integrating into the *MBS85* locus on Chromosome 19 in a site‐specific manner that requires the *AAVS1* genomic sequence. Superinfection with a helper virus reactivates AAV, allowing it to reproduce its genome and express its lytic stage proteins. Lysis of the cell by the helper virus helps AAV escape the cell.

Adeno‐associated virus is a non‐pathogenic commensal Parvovirus,^39^, ^40^, ^41^ whose biology makes it suitable as a gene therapy platform (Figure 4). It is unable to exit its latent stage and begin its lytic cycle spontaneously, without superinfection by another virus.^40^, ^41^ The natural AAV genome is capable of preferential site‐specific integration into the host genome at chromosome 19, but it can also maintain itself for long periods of time in the cell nucleus episomally (as an extra‐chromosomal element).^42^, ^43^, ^44^, ^45^, ^46^ There are 12 different natural AAV variants in humans (referred to as serotypes), each with a unique type of capsid which controls their tropism,^40^, ^47^ that is, the type of cells it can infect. Collectively, these variants confer upon AAV a very wide tropism, which can be further extended using non‐human and genetically engineered variants.^40^

An AAV vector is created by flanking a transgene expression cassette capable of producing the transcript of interest with viral sequences called inverted terminal repeats (ITRs).^40^ The ITRs allow the viral structural proteins to package the transgene cassette into virions. These engineered virions are typically produced by the expression of the ITR‐transgene cassette in cells that are also made to express the viral structural genes, from an expression vector rather than the viral backbone. Supplying the structural genes in trans, with only the ITR‐transgene available as the genome, allows packaging of the ITR‐transgene, without including any of the structural genes in the virion and therefore with a much lower risk of producing live virus in the process. By removing the structural genes, AAV vectors can transduce to transmit the transgene, but cannot replicate and create new virions. Typically, the genes needed for site‐specific integration of the AAV genome are not supplied during packaging and are not present in the vector. AAV vectors, therefore, lack the capacity for site‐specific integration and rely on episomal maintenance, substantially reducing their potential genotoxicity. The downside of episomal maintenance is rapid loss of the viral genome in replicating cells, limiting the utility of AAV to somatic post‐mitotic cells.^40^ An important constraint of AAV vectors is packaging capacity. AAV packaging has a size limit of approximately 5 kbp, this being at the low end of what viral vector systems can offer (i.e. lentiviral vectors can carry 8 kb inserts, and high‐capacity adenoviral vectors can include 37 kb). Considering all the elements (e.g. promoters, enhancers, regulatory domains) that need to be included, this is an important limitation. This size limitation is particularly salient for a second generation of AAV vectors that use the self‐complementary strategy, but we will discuss that along with an example of a self‐complementary AAV vector in Section 5.3. A key advantage of AAV vector systems is serotype switching, to alter vector tropism.^40^, ^48^, ^49^ Serotype switching involves packaging the vector with the capsid of the AAV variant most efficient at transducing the target cell population.

**FIGURE 5:**
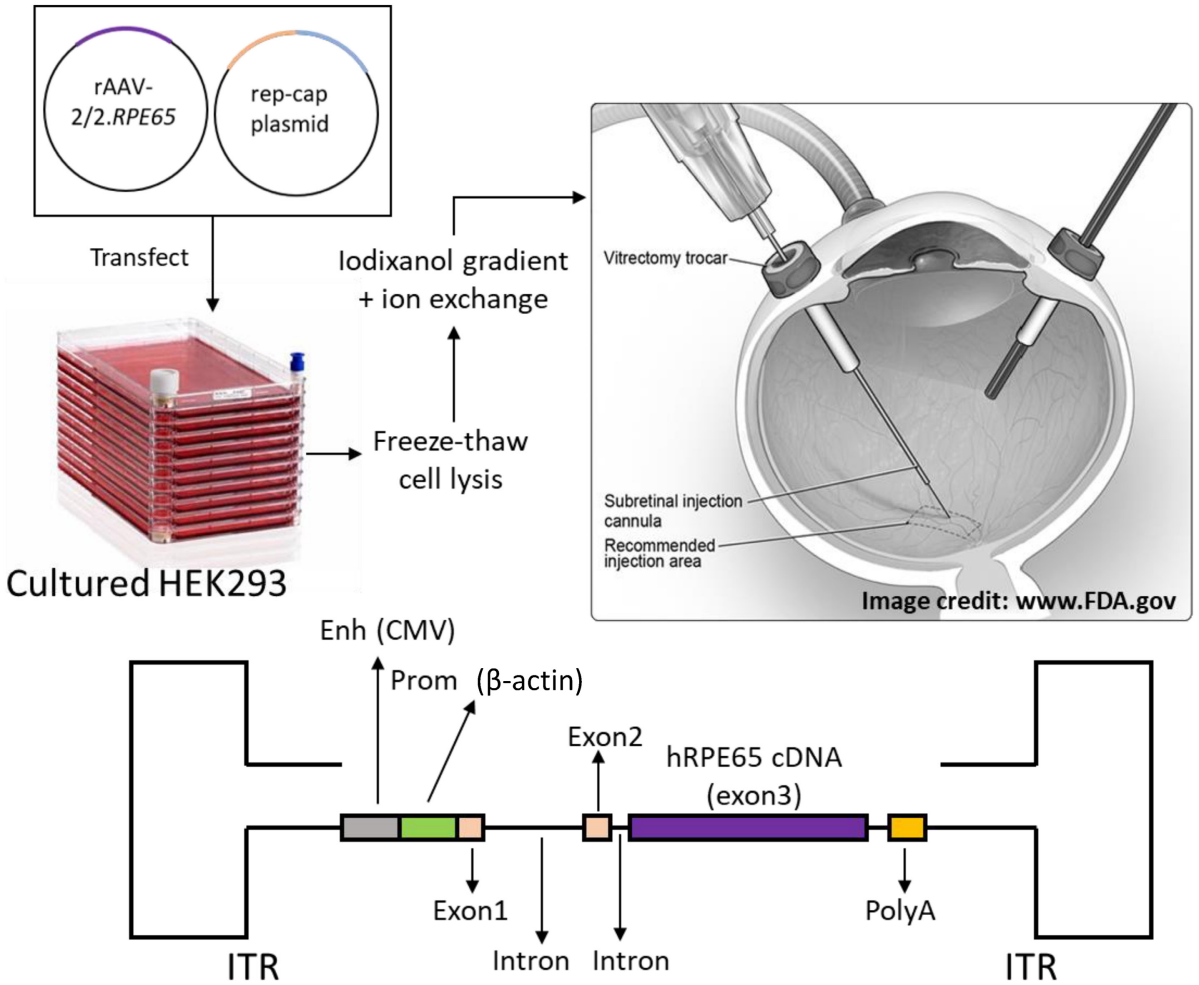
Luxturna vector design and treatment protocol. Luxturna is produced by packaging an expression cassette for *RPE65* into an AAV2 capsid. The *RPE65* vector virions are harvested from cells transfected with the relevant plasmids, purified and a high‐titre vector preparation is injected directly into the sub‐retinal space. The vector will diffuse outwards and insert the gene into a large region of the retinal pigment epithelium, creating a new source of 11‐cis‐retinal. The transcellular nature of the 11‐cis‐retinal cycle allows the effects to spread more widely throughout the retina. The cassette design contains the genomic *RPE65* sequence with all three exons and two introns. Efficient high‐level expression is ensured by an artificial promoter/enhancer pair created by joining the chicken β‐Actin promoter with the cytomegalovirus (CMV) enhancer and an artificial poly‐adenylation sequence. The cassette is flanked by the AAV2 ITR sequences.

Until very recently LCA2 was both incurable and untreatable. The approval of Luxturna has brought new hope, not just for LCA2 but also for a host of other previously incurable retinopathies. Luxturna is an AAV2‐based recombinant, non‐integrating vector designed to deliver the *RPE65* gene (Figure 5). In clinical trials, Luxturna was administered via subretinal injection into both eyes with a gap of 6–18 days.^33^, ^35^, ^36^, ^37^ Patients treated with Luxturna showed a strong and durable improvement in visual acuity 1 year after treatment. The visual field and the ability to perceive light also showed substantial improvements. Remarkably, patients from earlier phase 1 and 2 trials retained most of this improvement for 3 years or more. Such changes can have enormous effects on the quality of life of affected people, taking them from near blindness to partial sight.

The success of Luxturna has validated an entirely new therapeutic avenue for congenital retinopathies and other genetic afflictions of the eyes. Indeed, *RPE65* mutations account for only a small proportion of inherited retinopathies. Luxturna inspired an explosion in clinical development for gene therapy products targeting the eye.^34^ Outcomes should start filtering through in the mid to late 2020s.

### 2.2 Gene therapy for solid tumours: Imlygic and HSV gene supplementation for melanoma

Cancer is a genetic disease, whose extreme heterogeneity makes it a virtual microcosm for the RD field. Collectively cancer is common, but with so many different cancers, individual types can be rare. The highly variable ontogenesis and resistance to conventional treatments means personalized medicine is very challenging and yet also a key priority. Gene therapy offers an attractive proposition: taking advantage of specific defects within a particular cancer to create engineered viral vectors selectively toxic to that cancer. The technology can then be readily repurposed to target other cancer types.

**FIGURE 6:**
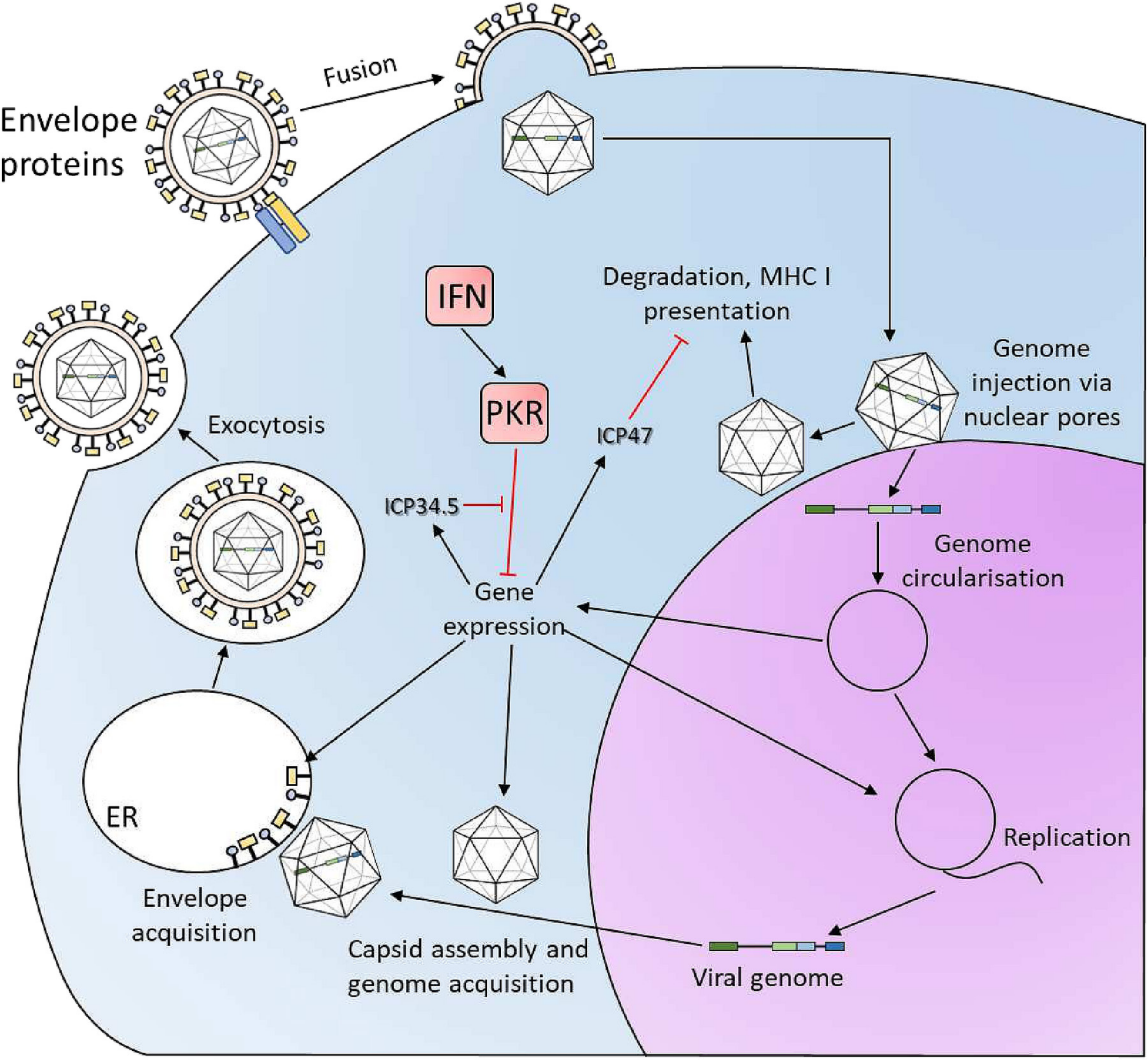
Herpes virus lifecycle. Herpes enter the cell by receptor‐mediated fusion of their envelope with the plasma membrane. The viral genome is injected into the nucleus, where it circularizes to facilitate simultaneous replication (through a rolling circle mechanism) and viral gene transcription. The linear viral genome copies are exported to the cytoplasm and interact with newly synthesized capsid proteins. The complete virion is internalized into vesicles within the endoplasmic reticulum (ER) guided by the viral envelope protein that is inserted into the ER membrane. The enveloped virus is trafficked within the ER into the endosomes and eventually released by exocytosis. Two viral proteins are important in blocking host responses against the virus. ICP47 blocks the loading of virion peptides onto MHC class I, hindering cytotoxic T‐cell recognition. ICP34.5 blocks protein kinase R (PKR), crippling the interferon pathway response to the virus.

**FIGURE 7:**
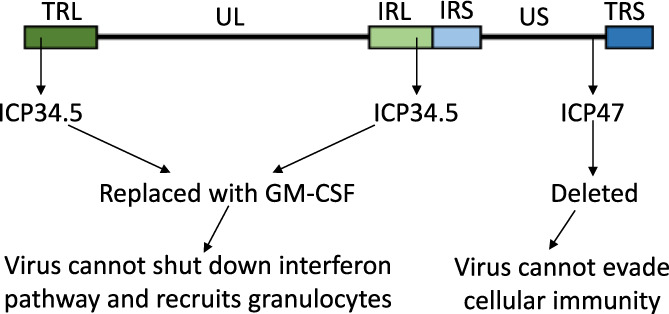
Talimogene laherparepvec (Imlygic) overall design. The herpes genome has two long inverted repeats (terminal and internal, TRL and IRL) and two inverted short repeats (IRS and TRS). It also has a long unique segment (UL) and short unique segment (US). In Imlygic, the promoters of the lytic cycle genes are altered for immediate activation, preventing lysogeny, *ICP34.5* is replaced with GM‐CSF and *ICP47* is deleted. Imlygic can only replicate effectively in cancer cells, where the interferon pathway and antigen presentation are compromised. Replication lyses the cancer cells and produces GM‐CSF, enhancing immune destruction of the tumour.

Melanoma is the fifth most common cancer in the United Kingdom, with an incidence of approximately 25 per 100,000.^50^ It is very aggressive, with in situ melanoma quickly progressing to metastatic disease, at which point survival rates drop precipitously.^50^ Talimogene laherparepvec (Imlygic) is a licenced herpes simplex virus‐1 (HSV1) gene therapy treatment for melanoma.^51^, ^52^ The lifecycle of herpes viruses is illustrated in Figure 6. Herpes viruses rely on key virulence factors that disrupt the interferon I pathway^53^ and antigen presentation to evade immunity^54^ and cause disease (Figure 6). In cancer, particularly melanoma, the same processes are often defective. Imlygic lacks these virulence factors, crippling its ability to infect normal cells, but leaving cancer cells highly vulnerable (Figure 7).^51^, ^55^ Two additional modifications enhance Imlygic's anti‐cancer potency: the virus expresses granulocyte‐macrophage colony‐stimulating factor (GM‐CSF) during replication, plus is unable to undergo lysogeny, immediately activating the lethal lytic cycle (Figure 7).^51^, ^55^

Imlygic performed well in clinical trials against stage III‐IV melanoma, refractory to surgery.^56^, ^57^, ^58^, ^59^ It increased the proportion of patients achieving durable disease remission, increasing disease‐free survival at 60 months by 50%. Although Imlygic was ineffective against late‐stage IV melanoma, it more than doubled overall survival in stage IIIB/B and IIIB‐IVM1. Remarkably, almost all patients achieving complete remission remained disease‐free at the 5‐year follow‐up. In addition, it was found that Imlygic shows substantial synergy with checkpoint inhibitors.^60^, ^61^

## 3 MODIFICATION OF DNA IN DIFFERENTIATED SOMATIC CELLS, PRIOR TO REIMPLANTATION

### 3.1 Ex vivo gene therapy benefits and challenges

Ex vivo gene therapy is the genetic modification of cells outside the body, followed by transplantation. These cells could be differentiated somatic cells or stem/progenitor cells.^62^ The main advantages of this ex vivo approach include the selective targeting of the cell population of interest, the avoidance of immune defences and the implementation of quality control systems before the genetically modified cells are reimplanted. In this section, we will focus on differentiated somatic cells that retain sufficient replicative capacity to allow extraction, modification outside the body and re‐implantation.

### 3.2 Retroviral vectors for ex vivo gene therapy: chimeric antigen receptor (CAR) T cells

**FIGURE 8:**
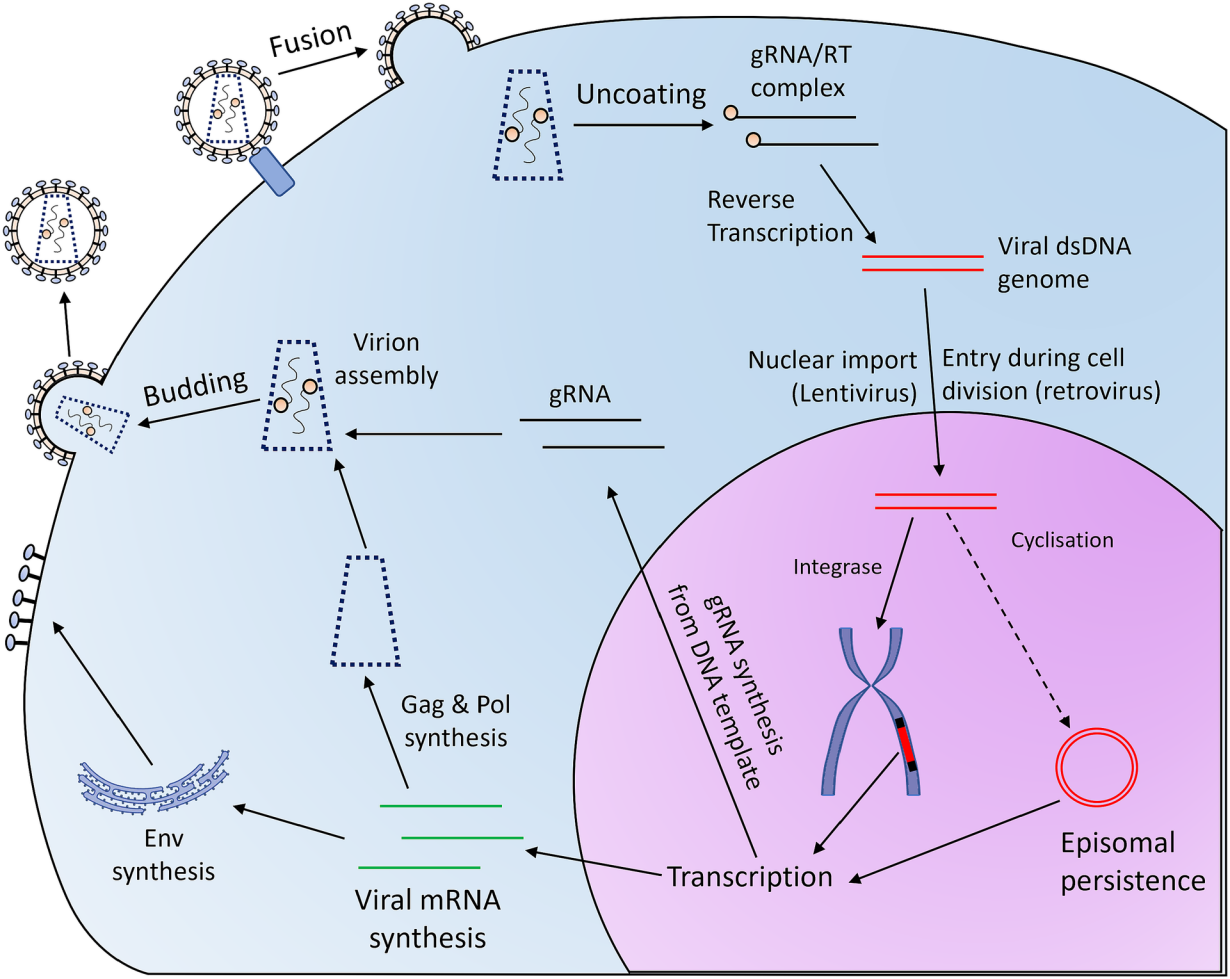
Retrovirus life cycle. Retroviruses and lentiviruses enter the cell via receptor‐mediated fusion of their envelope with the cell membrane. In the cytoplasm, the viral capsid disassembles and genomic RNA (gRNA) is converted to a double‐stranded (dsDNA) genome by reverse transcriptase (RT). The dsDNA genome is either transported through the nuclear pores by viral proteins (lentivirus) or enters the nucleus during cell division (gamma‐retrovirus). In the nucleus, cyclization and episomal persistence or direct integration into the host chromosomes occur. Integrated dsDNA produces both viral transcripts and the gRNA genome. The viral particles are assembled in the cytoplasm. The envelope protein, which is inserted in the plasma membrane, directs budding of the assembled nucleocapsid out of the cell and acquisition of the viral membrane envelope.

Retroviruses are enveloped single‐stranded RNA viruses, whose life cycle involves converting their RNA genome into double‐stranded DNA and stably integrating it into the host genome.^63^, ^64^, ^65^ Their RNA‐containing capsid is surrounded by a lipid bilayer derived from the host cell plasma membrane and containing the envelope protein, a transmembrane host cell invasion factor. Gammaretroviruses^66^ and Lentiviruses^67^ are most used as viral vectors. Figure 8 shows a brief summary of the retroviral/lentiviral life cycle. Retroviruses use special sequences called long terminal repeats (LTRs) to direct packaging of their genome into virions.^66^, ^67^ The LTRs contain signals facilitating several steps in the virus life cycle and act as powerful promoters. Retroviral vectors are made from an LTR‐flanked transgene cassette by supplying the virus structural proteins in trans.

Retrovirus vectors are thus used to directly insert transgenes into the host genome. Although integration is promiscuous, it is not random and integration patterns have been reported. For example, HIV prefers active transcriptional units but integrates at similar frequencies across the gene.^68^ Although the promiscuity of the integration is potentially problematic, it also ensures that the payload will be inserted in an area of active chromatin. Lentiviruses offer an additional advantage over other retroviruses. They can translocate their genome across the intact nuclear membrane and do not need to wait for cell division to access the host genome for integration. Lentivirus vectors can thus transduce quiescent cells.^67^

Acute lymphoblastic leukaemia (ALL) is a malignancy of the lymphocyte progenitor cells that primarily affects paediatric patients.^69^ It is associated with a range of chromosomal translocations in key oncogenes, such as *PAX5* and *TCF3* and, in some cases, gain of function mutants, such as the *BCR‐ABL1* (Philadelphia translocation) and *ETV6–RUNX1* rearrangements.^70^, ^71^, ^72^ The incidence of ALL is estimated at 10–20 cases per million.^69^ Unlike many other inherited cancers, ALL develops early in life making it one of the most common paediatric cancers. B‐cell precursor malignancies account for the bulk of ALL. Advances in chemotherapy and the understanding of the signalling networks that are disrupted by mutations in ALL, have led to the development of relatively effective chemotherapy regimens,^73^, ^74^, ^75^ but unfortunately, when patients relapse after treatment or even worse when initial responses are poor, the prognosis is typically bleak.^76^, ^77^

Non‐Hodgkin lymphoma (NHL) is one of the more common lymphoid tissue‐derived cancers affecting the head and neck.^78^, ^79^ It includes a highly heterogeneous group of malignancies that collectively have an incidence of approximately 1 in 10,000 in the European Union. Diffuse Large B‐cell Lymphoma (DLBCL) accounts for between a quarter and a third of NHL incidence and it is highly aggressive.^80^, ^81^ Familial forms of NHL have been characterized.^82^, ^83^ Like ALL, effective treatment options are generally available for NHL, but refractory DLBCL has a very poor prognosis.^80^

**FIGURE 9:**
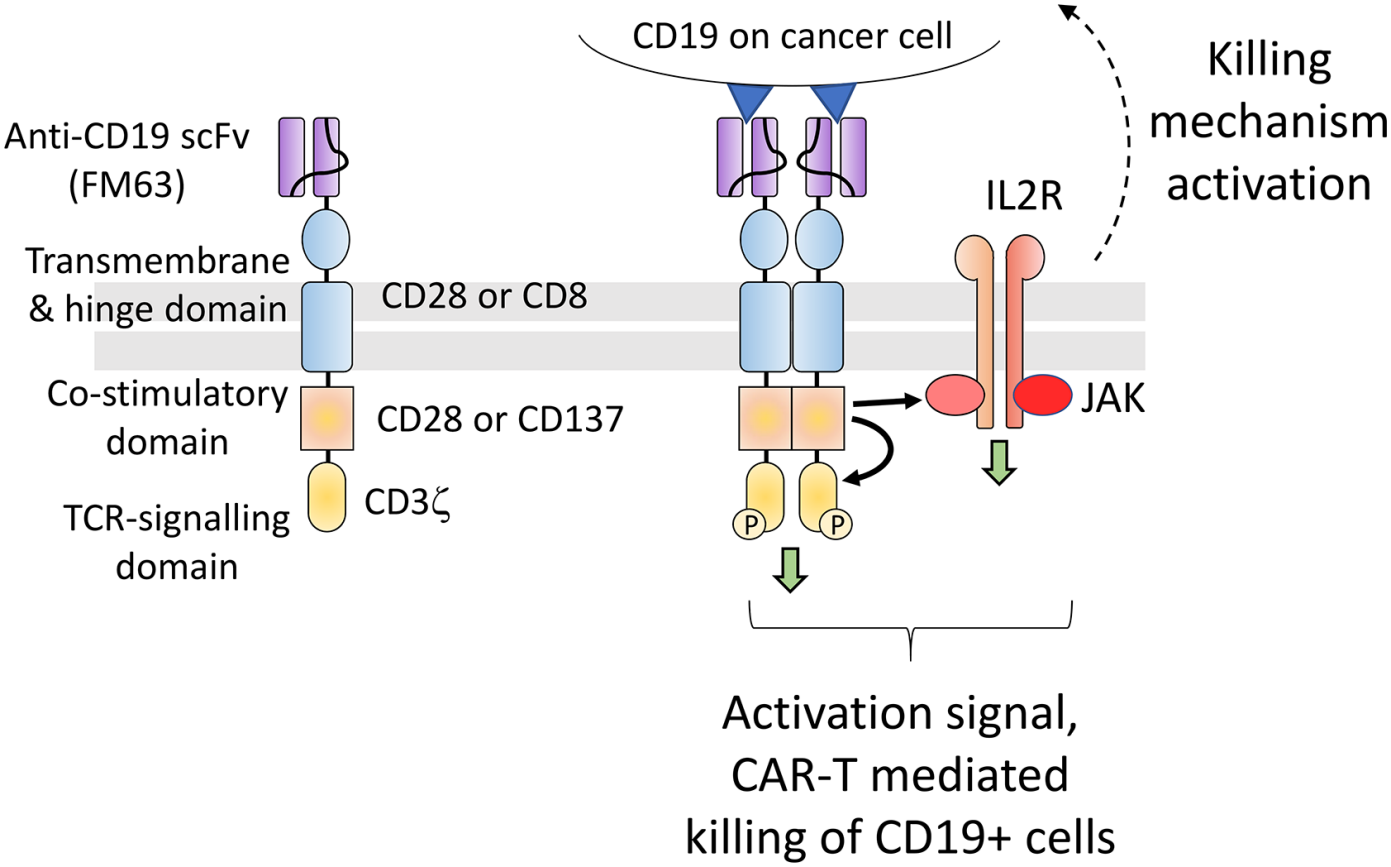
The chimeric antigen receptor (CAR) design. The artificial T‐cell receptor (TCR) is made in a modular manner by combining the following parts: the CD3ζ signalling domain from the TCR, a transmembrane and hinge region and co‐stimulatory domains from receptors that are needed for TCR signalling, such as CD8, CD28 and 4‐1BB (CD137), and a single chain variable fragment (scFv) targeted against the protein of interest. This creates a receptor capable of generating a full TCR signal upon binding of the scFv target. The CAR is thought to become activated by dimerization or multimerization allowing cross‐interaction between the signalling domain of one CAR molecule with the co‐stimulatory domain of another. This drives CAR activation, creating a CD3 signal and activating other accessory receptors, particularly the IL2 receptor.

The development of artificial T‐cell receptors (TCRs) which target CD19, a surface marker of B‐cell precursors that is strongly expressed in most B‐cell lymphomas,^84^, ^85^ was a revolutionary advance (Figure 9). These chimeric receptors are constructed by joining together the signalling apparatus of the TCR with parts taken from costimulatory molecules and a binding site against the target protein, in this case CD19.^86^, ^87^, ^88^, ^89^, ^90^ Anti‐CD19 CARs target T‐cells artificially to B cells. The CAR signalling steps are distinct from actual TCR signalling,^91^, ^92^, ^93^, ^94^, ^95^ vary depending on the type of costimulatory molecule used, and the immunological synapse formed is atypical. Nevertheless, the signals produced are sufficient to emulate T‐cell receptor signalling, enabling the modified T‐cells to attack and destroy targets expressing CD19 while bypassing the human leucocyte antigen (HLA) restriction.

Autologous CD19‐CAR T‐cells turned out to be highly effective in treating refractory NHL and ALL. The first two products approved for this purpose were tisagenlecleucel (Kymriah) and axicabtagene ciloleucel (Yescarta). Kymriah^88^, ^89^ is a preparation of CD19‐CAR engineered T‐cells using the 4‐1BB co‐stimulatory domain and the CD8alpha transmembrane and hinge region. CD3‐positive cells (CD4 and CD8 T‐cells) isolated from autologous blood are transduced, using a lentiviral vector, with a CD19‐CAR transgene driven by the Elongation Factor‐1 promoter.^88^ After selection and expansion, the CAR‐T cells are reinfused into patients that have undergone lymphocyte depletion treatment.^89^ Yescarta^96^, ^97^, ^98^, ^99^ is prepared in a similar manner, except it uses the CD28 transmembrane/hinge region and co‐stimulatory domain instead of CD8alpha/4‐1BB. The vector is Retroviral and driven by the mouse stem cell virus (MSCV) promoter.^99^

Kymriah is licenced for the treatment of refractory ALL and NHL/DLBCL, while Yescarta is only licenced for refractory NHL/DLBCL.^100^, ^101^ Both help drive substantial remission rates in excess of 50%, whereas conventional treatments are largely ineffective.^89^, ^96^ They can also have serious side‐effects.^102^, ^103^, ^104^, ^105^, ^106^ Cytokine release syndrome is a potentially life‐threatening complication capable of harming several major organs, including the nervous system via the IEC (immune effector cell) associated neurological syndrome or ICANS. A second distinct complication of CD19 cell elimination is predictably beta‐cell aplasia and a collapse in blood immunoglobulin levels. This can potentially predispose the patient to infections and must be managed to mitigate the risks.

The success of CAR‐T technology in blood cancers has spearheaded substantial research on possible applications to solid tumours, a more challenging environment. Applications of gene therapy to cancer now account for around half the gene therapy treatments under development.^107^

## 4 MODIFICATION OF DNA IN STEM CELLS, PRIOR TO REIMPLANTATION

An alternative ex vivo approach targets stem/progenitor cells,^62^ which underpin the natural maintenance of organs. This strategy is particularly suited to correction of genetic defects of the blood, which builds on the clinical experience of bone marrow transplantation.^108^, ^109^, ^110^, ^111^

To illustrate the effectiveness of this approach, we will look at two clinically approved treatments for genetic diseases affecting blood cells, Strimvelis for ADA‐SCID and Zynteglo for β‐thalassaemia.

### 4.1 Severe combined immunodeficiency: Strimvelis HSC gene therapy

Severe combined immunodeficiency is a heterogenous group of genetic disorders that cause complete or nearly complete impairment of T‐lymphocyte function, combined with primary or secondary dysfunction in other immune cell types.^112^ The SCID spectrum is very rare, with a prevalence of approximately 1 in 60,000 live births.^113^ The complexity of T‐lymphocyte ontogenesis explains the extensive genetic heterogeneity of SCID. There are currently 16 known causative genes and over 20 separate defects.^114^, ^115^ The most common mutations are in X‐linked IL2 receptor components (SCID‐X1).

Adenosine deaminase‐severe combined immunodeficiency is another common form and one of the most damaging.^116^ The Adenosine Deaminase enzyme is essential for the purine salvage pathway that regulates the purine nucleotide balance. ADA activity is important in preventing adenine nucleotide accumulation. Lack of ADA results in a marked imbalance in the dNTP pool, compromising DNA polymerase function.^117^, ^118^, ^119^ In rapidly or continuously proliferating cells the result is genotoxic shock and apoptosis,^120^, ^121^ and the lymphoid cell differentiation pathway is particularly sensitive to ADA deficiency.^116^, ^122^, ^123^ ADA deficiency also impacts cAMP synthesis, disrupting general cell signalling and giving rise to a more diffuse pathology, in most other tissues including the brain.^124^

Adenosine deaminase‐severe combined immunodeficiency can be treated by allogeneic haematopoietic stem cell (HSC) transplantation^10^, ^125^ and PEGylated ADA (PEG‐ADA).^10^, ^126^ PEG‐ADA has a long plasma half‐life and can help reduce intracellular adenine build‐up, by keeping extracellular levels low and facilitating transporter‐mediated efflux, alleviating some of the worst symptoms.^10^ Bone marrow transplantation is limited by the availability of HLA‐matched donors and by the risk of graft‐versus‐host disease (GVHD) with allogeneic donors.^127^

A gene therapy option for ADA‐SCID has been licenced by the European Medicines Agency (EMA) in the European Union (EU). Strimvelis is a preparation of autologous HSCs, engineered to express functional ADA.^128^, ^129^, ^130^, ^131^ CD34‐positive HSC cells are isolated from the person affected and expanded using a cocktail of soluble mediators: FLT3L, KITL/SCF, THPO, IL3, and IL6 (FKT36). In this proliferative state, the cells are transduced with a functional ADA copy using an amphotropic Murine Moloney Leukaemia virus vector,^132^ whose 4070A envelope gene, targets Pit‐2 and mediates efficient transduction of HSCs.^133^ The transduced HSC pool is re‐infused after non‐myeloablative conditioning with anti‐proliferative agents such as busulfan to suppress the proliferation of endogenous HSCs.

Strimvelis has performed exceptionally well in clinical trials.^131^, ^134^, ^135^ A follow‐up of 18 people treated with Strimvelis at a very early age revealed that all of them survived (follow‐up 2–13 years, median 7 years), and they were well enough to resume normal social interactions. Several of the patients were able to return to school. In those who could be evaluated ADA expression reached or exceeded 10% normal and remained stable in all myeloid and lymphoid cells, immune function was successfully reconstituted and a response to antigen challenge could be observed. The rate of infection decreased dramatically, and the recipients managed to resolve infections in most cases. In almost all cases PEG‐ADA treatment could be discontinued. Intervention‐free survival remained above 80%. These results match autologous HSC transplantation and compete very favourably with all other treatments.

Successful reconstitution of the T‐cell population does not eliminate the complete health impact of ADA‐SCID, since it does not replace ADA function in cells of a non‐haematopoietic linage, but it effectively provides (via expression in red blood cells) a ready pool of plasma ADA that can serve the same function as PEG‐ADA. At the same time, it eliminates the supply issues with donor‐matched HSC transplantation and the risks associated with allogeneic transplantation (e.g. GvHD).

### 4.2 β‐Thalassaemia gene therapy with Zynteglo HSCs

Beta‐thalassaemia is one of the most common genetic anaemias.^136^, ^137^ It is autosomal recessive, with a highly variable distribution. Its prevalence approaches 1 in 1000 live births in areas where malaria is currently endemic or was endemic in the recent past but is very rare elsewhere. Globally, prevalence is close to 1:100,000 live births.

The disease phenotype depends on the exact genetic defect in the *HBB* (adult β‐globin) gene, a subunit of haemoglobin. Homozygous inheritance of an allele that produces no functional protein causes β‐thalassaemia major and severe life‐threatening anaemia.^136^, ^137^ Homozygous inheritance of a partial loss of function mutant leads to β‐thalassaemia intermedia and milder disease.

Beta‐thalassaemia has all the typical hallmarks of anaemia^136^, ^137^ including fatigue, weakness, and palpitations. The major disease also leads to muscle cachexia, skeletal and cartilage deformities, osteoporosis and splenomegaly. Regular blood transfusions can address most of these symptoms, but they generally also cause iron overload, leading to heart, liver and endocrine complications.

Recently an ex vivo gene therapy approach, Zynteglo^138^, ^139^, ^140^, ^141^ was licenced for the treatment of severe, transfusion‐dependent, β‐thalassaemia. The patient is treated with G‐CSF and a CXCR4/SDF‐1 antagonist, which leads to substantial proliferation and mobilization of HSCs from the bone marrow into the blood. CD34‐positive HSCs are collected from the blood and transduced in the laboratory with the BB305 lentiviral vector, which contains a mutated *HBB* (T87Q).^142^, ^143^ The BB305 lentiviral vector has a self‐inactivating design, which removes the transcriptional activity of the LTR. It includes the entire *HBB* coding sequence with its native control elements: the β‐globin promoter,^144^ its 3′ enhancer^145^ and selected fragments from the upstream locus control region,^146^ facilitating high‐level expression. The T87Q variant confers enhanced anti‐sickling activity and can be differentiated chromatographically, serving as a biomarker.^147^ The patient is conditioned with myelosuppressive drugs to facilitate donor cell engraftment prior to infusion of the corrected HSCs.

Like Strimvelis, Zynteglo was highly successful in clinical trials.^138^, ^139^, ^140^, ^141^ The majority of patients showed long‐lasting improvement in haemoglobin levels and were able to stop blood transfusions. Most patients that could be evaluated for over a year, achieved near normalization of haemoglobin levels and blood transfusion independence.

## 5 GENE THERAPY THROUGH MANIPULATION OF POST‐TRANSCRIPTIONAL RNA PROCESSING AND TRANSLATION

**FIGURE 10:**
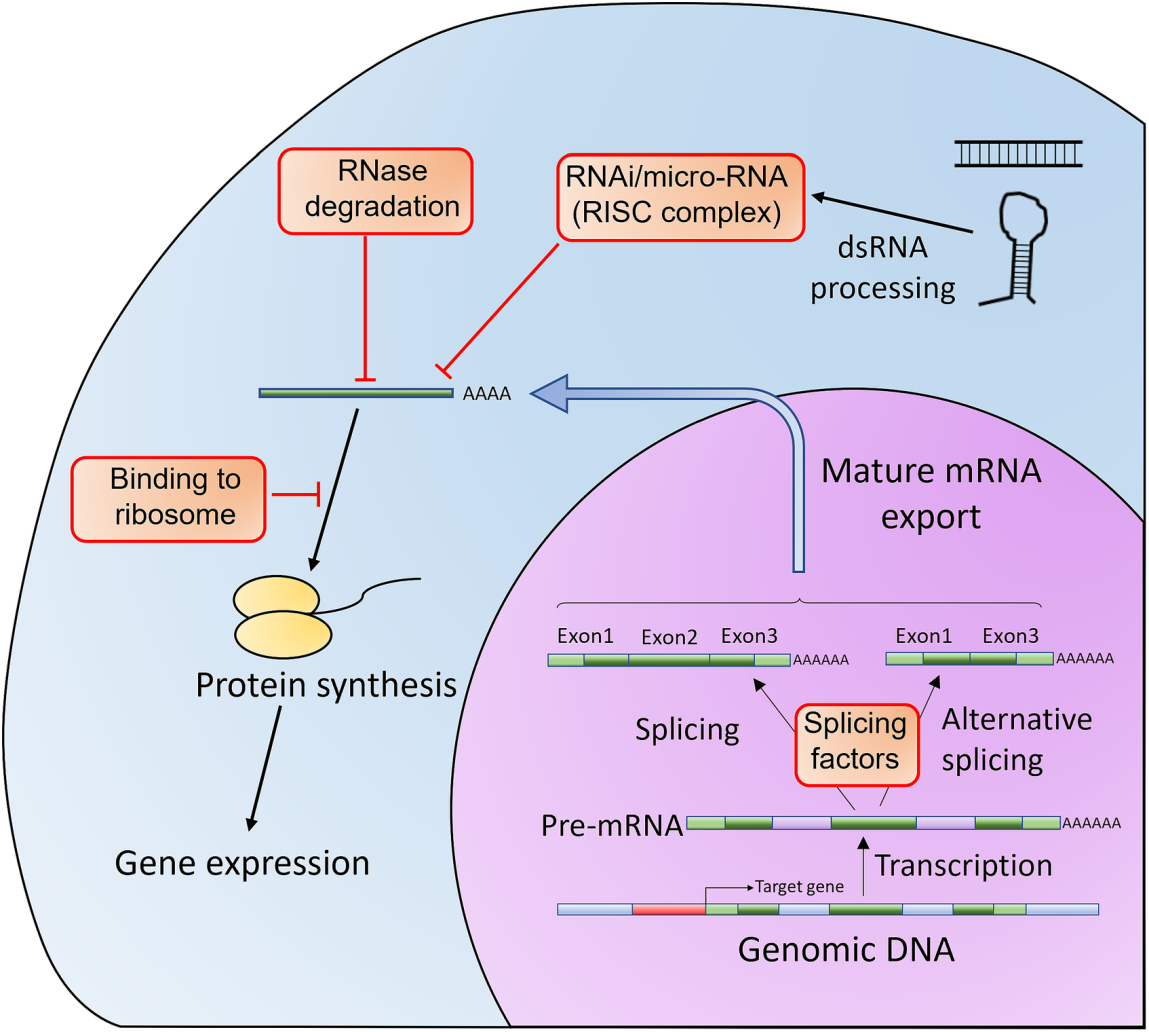
Post‐transcriptional control of gene expression in eukaryotic cells. The first control point is during splicing. Splicing in eukaryotic cells is controlled by a series of splicing site elements and factors, and more than one product can be produced from the same gene. The splicing factors expressed by the cell determine the splice variant balance. The mature RNA is further regulated by degradation. A special protein complex recognizes double‐stranded RNA and then uses it as a template to degrade matching mRNA molecules. This mechanism (RNA interference) allows the cell to fine‐tune gene expression, by producing special RNA molecules (micro‐RNAs or miRNA). Splicing and RNA interference can be controlled using artificial oligonucleotides. The final control point is binding to the ribosome and translation initiation.

Gene expression levels can be modulated after transcription using synthetic nucleic acid molecules able to interfere with splicing, translation or RNA degradation, without directly altering the cell's genetic material (Figure 10).^148^ Diseases resulting from gain‐of‐function mutants are particularly amenable to this intervention method.

**FIGURE 11:**
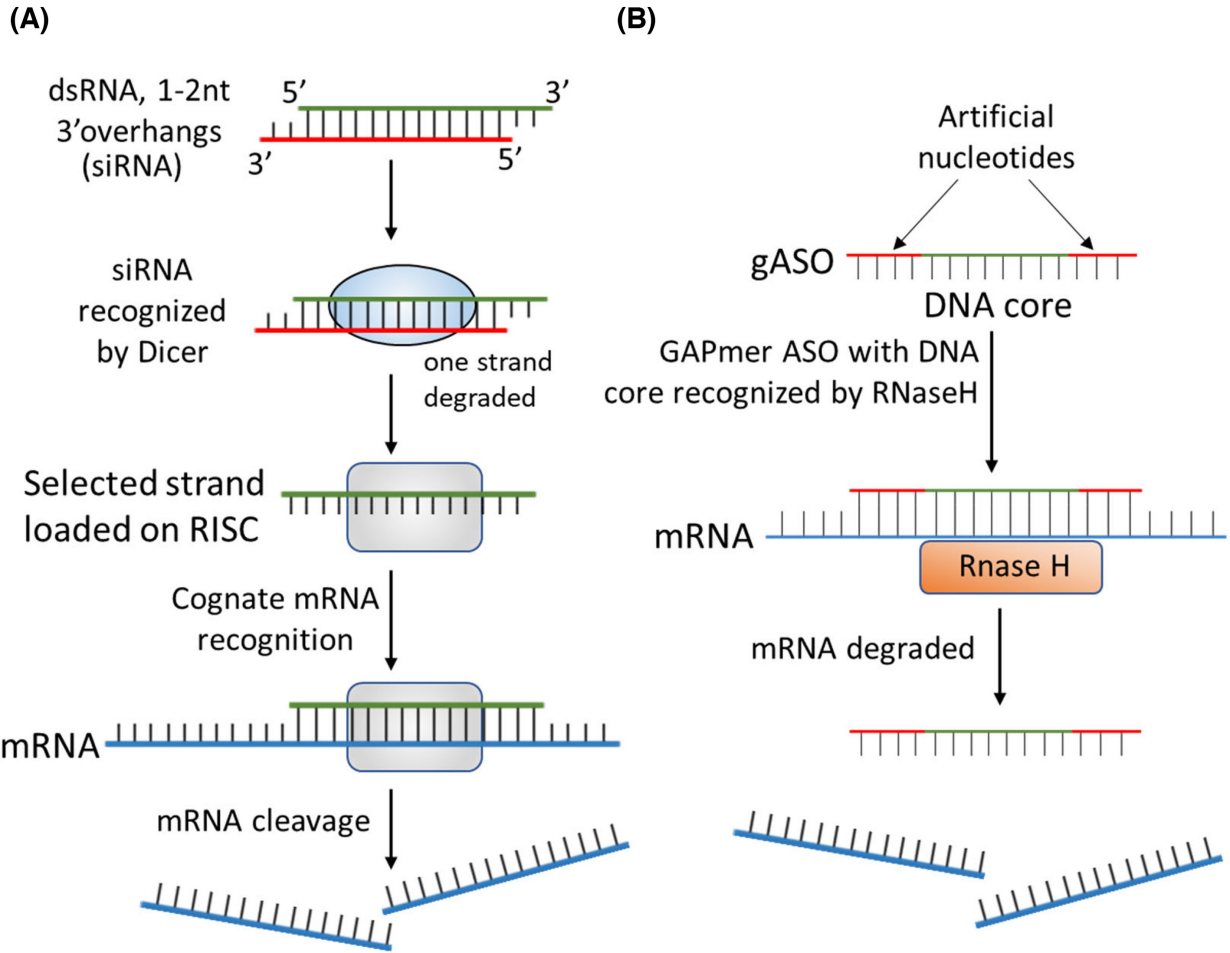
Oligonucleotide control of gene expression. (A) siRNAs are duplex RNA molecules made from two complementary 20–21 nt strands designed to leave 1–2 nt overhangs on the 3′ side after annealing. The Dicer protein complex processes siRNAs and incorporates one of the strands and degrades the other based on their physical properties. Any mRNA sequence that can base pair with the chosen strand is degraded. Artificial siRNA molecules are designed to force selection of the non‐coding strand. (B) Another design is gapmer antisense oligonucleotides (gASOs), which consist of a DNA core flanked by artificial nucleotides. The artificial nucleotides are nuclease resistant and have a high affinity for RNA. When the gapmer ASO anneals to its target RNA, the DNA core forms a DNA/RNA heteroduplex, thus recruiting RNaseH and marking the target RNA for degradation. The resistance of gASOs to nucleases allows for cytoplasmic persistence and durable responses.

Control of RNA levels within the cell occurs through RNA interference,^148^, ^149^, ^150^ which uses endogenous (e.g. miRNA) or exogenous (e.g. siRNA) double‐stranded RNA templates, to target specific mRNA sequences for degradation. Artificially produced RNA molecules (short hairpin RNAs, which are artificial miRNA mimics^151^, ^152^) can be used to hijack this system and selectively target mRNA molecules for degradation^153^, ^154^ (Figure 11).

Antisense oligonucleotides (ASO) are short nucleic acid sequences designed to base pair with a specific RNA target within the cell.^148^ Typically, ASOs consist of modified nucleotides with increased stability, and frequently include artificial nucleotide analogues, such as morpholinos^155^ and locked/bridged nucleic acids.^156^ ASOs can manipulate the post‐transcriptional fate of mRNA in various ways,^157^ but here we will mostly focus on RNase H targeting (Figure 11). Splice‐switching oligonucleotides (SSO) are designed to base pair with splicing sites, or splicing enhancers/suppressors within a pre‐mRNA sequence and direct alternative splicing of the target gene.^158^ RNA ASOs designed to base pair with sequences within the 5′ or 3′ untranslated region (UTR) can suppress or enhance mRNA translation.^159^, ^160^

Here, we will discuss some key examples of RNA interference and SSO‐based therapeutics that have recently cleared clinical trials and are now being used to treat rare diseases. In particular, we will look at familial transthyretin amyloidosis (FTA) and spinal muscular atrophy (SMA).

### 5.1 Familial transthyretin amyloidosis: Onpattro RNAi and Tegsedi ASO

Familial transthyretin amyloidosis is a rare genetic disease of the Transthyretin (*TTR*) gene that causes the protein to misfold.^161^, ^162^, ^163^ The misfolded protein forms amyloids that deposit into and damage tissues. This is a slow gradual process, so the symptoms typically begin in adulthood. The exact onset age is variable and correlates with disease progression. The peripheral nervous system is particularly vulnerable, so neuropathies are among the earlier symptoms, but as the disease progresses, eyes, kidney, heart, and CNS typically become involved. FTA is eventually fatal on average 10 years after the onset of symptoms, with a younger onset being associated with more aggressive disease. The prognosis in patients presenting with early cardiac involvement is extremely poor. Few patients survive for longer than 5 years.

Genetically, FTA mutations are autosomal dominant, but progression and penetrance vary depending on the exact genetic defect.^161^ Most patients are heterozygotes. The global prevalence of FTA is of the order of 1 in 10,000,^164^ though clusters have been observed within certain ethnic groups or populations, such as in certain areas of Portugal, Sweden, Japan, and West Africa.^162^, ^164^

The current gold standard treatment for FTA is liver transplantation^161^, ^162^, ^163^ since the liver is a major source of TTR. Liver transplantation arrests the development of polyneuropathies and slows but does not prevent progressive degeneration of the eyes, heart, and kidney.

In the last few years, the FDA has approved two oligonucleotide‐based therapeutics for FTA: Patisiran (Onpattro)^165^ and Inotersen (Tegsedi).^166^ Onpattro^167^, ^168^ is a stable nucleic acid lipid particle (SNALP) formulation containing short interfering RNA (siRNA—Figure 11) against TTR. Onpattro is a new generation of siRNA, using DNA overhangs (dTdT), instead of RNA (UU), increasing RNA resistance for a longer lasting effect. In addition, Onpattro has most of the U and C residues methylated in the sense strand, to promote incorporation of the siRNA correct strand into the RISC (RNA‐induced silencing complex) assembly. Onpattro is designed to target the 3′ UTR of the TTR transcript and will suppress expression of both mutant and wild‐type forms. This is desirable because once misfolded aggregates are formed, they can induce misfolding and deposition of even the wild‐type protein.

The Onpattro SNALP's formulation^169^ consists of a 1:1 mixture of cholesterol and phospholipids. The phospholipids have a strong positive charge (4:1 ratio of cationic to neutral) to facilitate complex formation with the siRNA. 5% of the cationic phospholipids used have a PEG2000 (polyethylene glycol 2000 MW) polymer attached to them, creating a sheath that greatly prolongs SNALP plasma half‐life. Lipid nanoparticles in the plasma are typically decorated by ApoE, despite the PEG sheath, and only extravasate effectively in tissues with fenestrated endothelium^170^, ^171^; thus, they naturally target the liver. After ApoE‐directed internalization, charged interaction between the positive SNALP and negatively charged endosomal membrane, mediates endosome escape, delivering the siRNA to the cytoplasm. The long SNALP half‐life, and the longevity of the primed RISC assembly, allow the effect to persist over several days.

Onpattro is highly effective at suppressing TTR expression. During clinical trials,^167^, ^168^, ^172^ it was found that expression is reduced by >70% within 5 days and remains below that threshold for at least 20 days. Infusion of Patisiran every 3 weeks over 18 months halted progression in virtually all patients that achieved sustained TTR suppression. Small but significant improvements were also seen in the polyneuropathy and cardiomyopathy aspects of the disease. Adverse reactions to Onpattro are primarily related to infusion of the liposomal formulation. Serious adverse effects were rare.

Tegsedi^173^, ^174^ is an RNaseH‐dependent GAPmer ASO formulation (Figure 11) also targeting the TTR 3′UTR. The inner DNA core has phosphorothioate linkages^175^ that make it strongly resistant to degradation but can also cause toxicity.^176^, ^177^, ^178^ To mask the phosphorothioate toxicity, the central core is flanked on either side by five 2′‐*O*‐methoxyethyl ribonucleotide residues, which are also resistant to degradation.^174^ The entire 20‐mer oligonucleotide is complementary to the target sequence in the TTR 3′UTR and stable enough to deliver via intramuscular injection of a preparation in saline, without a liposomal formulation. The ASO makes its way into the circulation and is actively taken up by cells in various tissues, with the liver being a primary site. During clinical trials,^173^, ^174^ patients received three injections of Inotersen in the first week, followed by weekly injections for a period of 64 weeks. At the end of the first‐week plasma TTR levels reduced by ~70% and remained at that level for the entire 64‐week period. Like Onpattro, Tegsedi effectively halted disease progression over the entire treatment period, albeit with variations between different patient groups.

The ability to control gene expression levels is vital in shutting down gain‐of‐function mutants. Like other nucleic‐based therapies, once the delivery method is optimized, it can be repurposed for any payload. For example, research into liposomal siRNA delivery paved the way for the SARS‐CoV2 mRNA vaccines, by BioNTech and Moderna.^179^, ^180^

### 5.2 Spinal muscular atrophy: Spinraza splice‐switching oligonucleotide

Spinal muscular atrophy is the most common cause of genetic death in childhood with a prevalence of approximately 1 in 10,000 live births.^181^, ^182^ It is caused by loss‐of‐function mutations in the survival motor neuron 1 (*SMN1*) gene.^183^ It is autosomal recessive and the severity of the phenotype inversely correlates with the copy number and expression level of the highly related, but only partially functional, *SMN2* gene.^184^, ^185^, ^186^ SMA is a systemic disease, due to SMN being ubiquitously expressed, but the lower motor neurons are particularly sensitive to loss of SMN function.^187^

*SMN2* differs by a few nucleotides from *SMN1*.^181^, ^184^, ^188^ Crucially, it is spliced differently, with 85%–90% of the transcripts typically skipping exon 7. The truncated protein isoform is unstable and rapidly degraded. *SMN2* proved to be pivotal in developing an oligonucleotide‐based therapeutic for SMA. Nusinersen (Spinraza) is an SSO designed to prevent the excision of exon 7 from the *SMN2* gene product, increasing the production of full‐length, stable SMN protein from it.^189^ The 20‐mer SSO oligo is made from 2′‐*O*‐methoxyethyl (2MOE) ribonucleotides,^190^ which resist degradation and base‐pair more efficiently,^191^ enhancing intronic splicing silencer‐N1 (ISS‐N1) inhibition. The oligo is injected directly into the spinal cord via lumbar puncture.^192^ Initially, a small number of frequent injections are given to quickly establish a steady state, followed by maintenance doses that are more infrequent. In the most recent clinical trial^192^ the conditioning regime is three bi‐weekly doses, followed by a maintenance regime with 4 months between doses. Spinraza has proven highly efficacious in infantile‐onset SMA (type I),^193^, ^194^, ^195^ producing dramatic improvements in survival and motor milestone achievement, with some infants developing skills never seen in the natural history of this disease. The incidence of adverse events was high, but mostly related to the complex spinal injection procedure in this vulnerable patient population.

Onpattro, Tegsedi, Spinraza and other examples represent important milestones for the oligo therapeutics field, by demonstrating that it is possible to exert sustained, effective, direct control over gene expression without stable genetic modification.

### 5.3 A gene therapy comparison of oligonucleotides versus viral vectors: Spinraza versus Zolgensma

**FIGURE 12:**
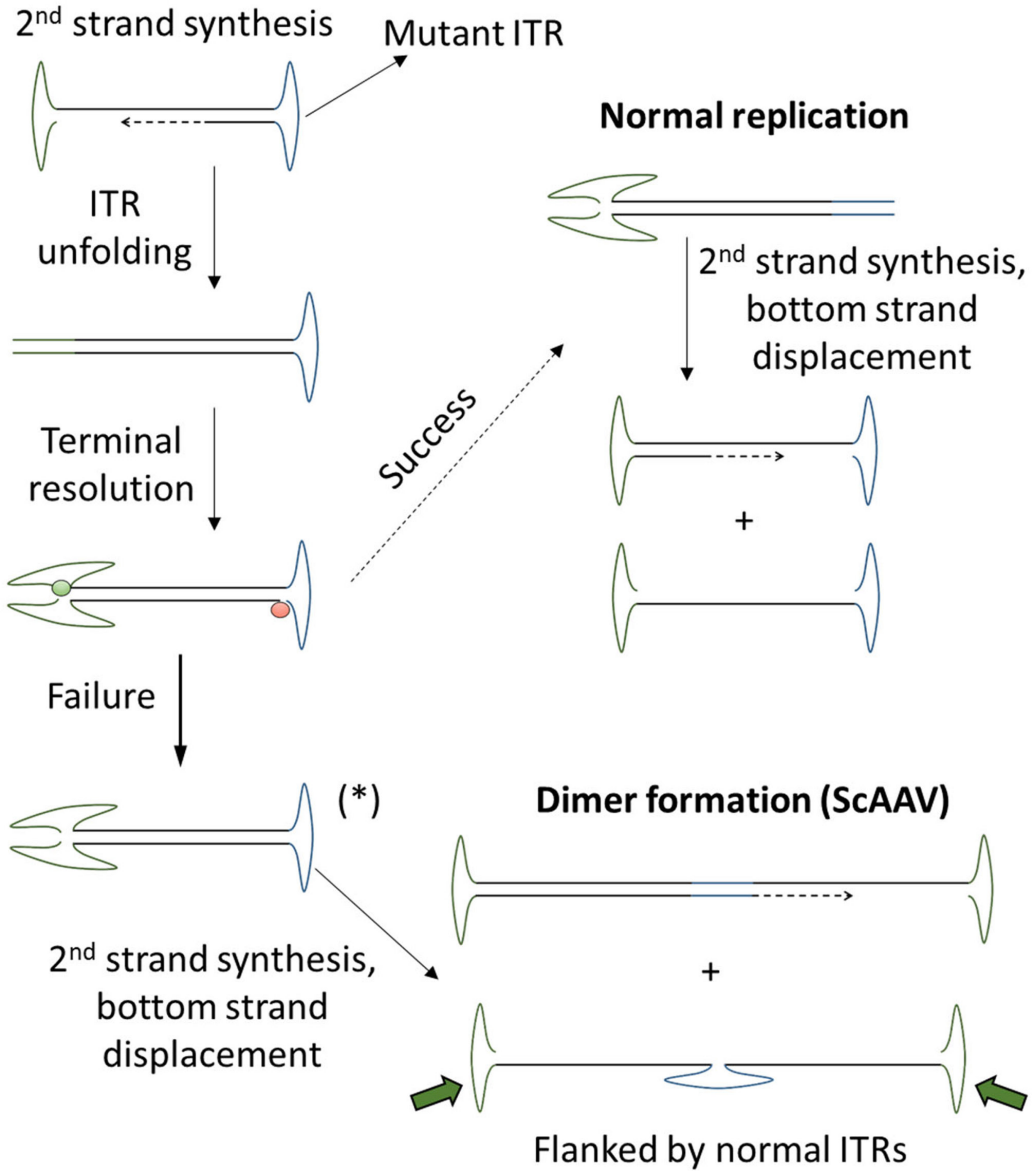
Generation of self‐complementary AAV vectors. AAV variants with faster expression kinetics can be produced through the use of a mutated ITR (blue, right), to frustrate terminal resolution. As a result, the two strands fail to separate, leaving them joined through the mutant ITR. The complete viral genome is still flanked by normal ITRs, therefore it can be replicated and packaged. Once released in the host cell the two strands can reanneal to form the structure marked with (*). This structure does not rely on second‐strand synthesis to stabilize it within the cell and initiate transgenic expression.

The existence of a viral vector gene therapy alternative to Spinraza offers a unique opportunity to directly compare the effectiveness of the two approaches. Zolgensma (Onasemnogene Abeparvovec)^196^, ^197^, ^198^, ^199^, ^200^ is an AAV‐based gene supplementation treatment aimed at directly and permanently restoring *SMN1* expression with a single dose. The design of the Zolgensma expression cassette is similar to Luxturna (Figure 5), in using the hybrid CMV–Chicken beta actin promoter to drive the expression of *SMN1* cDNA. To enhance expression, the design incorporates an artificial intron (from SV40) and codon optimization. The sequence of AVXS‐101 (the vector for Zolgensma) is proprietary and the exact optimizations are not in the public domain, but the effectiveness of this approach was documented by using a similar AAV9 platform.^201^, ^202^, ^203^ A self‐complementary design (Figure 12) was employed, where one of the flanking ITRs was a specially engineered variant to synthesize genome dimers, rather than monomers.^204^ This design is advantageous in that it can speed up transgenic expression without the need for DNA synthesis, a possible rate‐limiting step for single‐stranded AAV vectors.

Zolgensma performed very well in clinical trials,^196^, ^197^, ^198^, ^199^, ^200^, ^205^ showing both a high response rate and substantial symptom alleviation. In the SPR1NT phase 3 trial, all 14 participants achieved the primary endpoint of sitting unaided, while 11 and 10 out of 14 managed to stand and walk respectively. None of the 23 untreated SMA patients achieved these developmental milestones. Impressively, a substantial number of children (40%–80% depending on the endpoint), reached the milestones within the regular developmental time. Motor assessment showed that all children improved rapidly after administration and reached at least 80% of the normal score. Typically, SMA children achieve on average 40% of the normal score and their scores decline, rather than improve with age. All children managed to avoid the need for mechanical ventilation during the study and 13 children were spared the need for assisted feeding. This was confirmed by other studies, which also found that the gains were durable. These results compare favourably with Spinraza. Indeed, Bitetti et al.^205^ investigated children previously treated with Spinraza and found that Zolgensma helped the children make further gains, with the greatest benefits in children that had responded less well to Spinraza. An important factor in this improved response is likely to be the systemic treatment provided by Zolgensma, while Spinraza is delivered by intrathecal injection, with direct beneficial effects expected to be restricted to the CNS.

Although the evidence so far points to a higher efficacy and response rate for Zolgensma compared with Spinraza, safety concerns with the use of AAV9 use have emerged. Two common serious adverse events have been observed, hepatotoxicity and thrombocytopenia. Although these adverse effects proved self‐limiting in the clinical trials, a subsequent meta‐analysis^206^ of the clinical data confirmed that the majority of patients show evidence of liver damage, though this responded well to steroid treatment.

In 2021, Thomsen et al.^207^ reported the expression of *SMN1* in two infants that received Zolgensma but had died due to reasons unrelated to treatment. *SMN1* expression was readily observed in the central nervous system, but also in several peripheral organs, particularly the liver where expression was 2–3 orders of magnitude higher compared to the CNS. Although the reasons for AAV9 vector hepatotoxicity are not fully understood, the observation that it responded to steroid treatment suggests that it might be related to an immune response to the vector. Sadly, two patients recently treated with Zolgensma have died of acute liver failure. Both deaths occurred several weeks post‐treatment, shortly after corticosteroid taper was initiated.^208^ Clinical trials with AAV vectors for other indications have also been marred by similar severe adverse events to those described for Zolgensma, including death at high vector doses. While Zolgensma has been used on more than 2300 people so far, these findings reiterate the need for better understanding and control of viral vector tropism and the associated immune response, to develop even safer treatments.

Just as the lessons learned from Luxturna are expected to greatly reduce the effort required to target retinopathies, so will Zolgensma aid the development of other gene therapy solutions targeting the central nervous system,^209^ thereby offering further evidence for the suitability of gene therapy to treat RDs. Moreover, as development and production costs fall with the adoption of these methods into mainstream clinical practice, we can expect treatment costs to reduce significantly from their current high price tags.

## 6 CONCLUDING REMARKS AND FUTURE PROSPECTS

In this review, we have used specific examples of successful clinical implementation to showcase what gene therapy can achieve and how it is already helping address the challenges of treating RDs. Around 50 years have passed since gene therapy was first mooted as a possible therapeutic avenue, illustrating how complicated it can be to implement a novel concept into clinical practice. Some of the licenced therapies fit the original mould (gene supplementation to rescue a genetic defect), but many do not as they involve mechanistic pathways discovered and adapted more recently, such as dsRNA interference, splicing modulation and exon skipping. Significant initial successes in treating SCID with ex vivo gene therapies built on the accumulated experience of allogeneic bone marrow transplantation, showed how existing clinical practice can be instrumental for implementation of novel technologies. Application of gene therapies to some forms of SCID paved the way to develop clinical gene therapies for other immunodeficiencies, and also additional disorders of the haematopoietic system like β‐thalassaemia, thus demonstrating how therapeutic strategies can be adapted relatively quickly to different diseases of the same tissue. Moreover, advances in ex vivo modification of haematopoietic cells have also led to unforeseen successes such as CAR‐T cells. A similar expansion is underway with AAV9 vectors, which can cross the blood–brain barrier via intravascular delivery to treat inherited diseases of the CNS, as first demonstrated with Zolgensma for SMA.^196^, ^197^, ^198^, ^199^, ^200^ For those interested in tracking marketed gene (and cell) therapies across the world, the International Society for Stem Cell Research maintains an up‐to‐date map.^210^ Note, however, that this resource does not include oligonucleotide therapies, as they are not technically considered gene therapies by FDA or Advanced Therapeutic Medicinal Products by EMA.

The application of gene therapy technologies to the vaccine field has provided resounding successes in the fight against COVID‐19, with mRNA‐based and adenovirus‐based formulations being developed in record time and used to immunize a large part of the world population. These platforms are now being explored for other applications in both vaccinology and RD therapy, with very promising prospects.

Our discussion has focused on some of the clinical successes of gene therapy. Consequently, we have not dwelt on other recent advances in gene therapy methods that are yet to reach full clinical implementation. However, to conclude our review we briefly cite some recent advances likely to drive the field forward. These include synthetic virology,^211^ lipid nanoparticles^212^ and membrane‐active peptides^213^ as key areas of intense research development. Similarly, AAV capsid engineering to alter tropism has seen much resource investment and is starting to deliver optimized AAV serotypes for targeted therapies in vivo. However, to close this review it is clear that genome editing technologies are easily the most promising therapeutic strategies for the future. The accessible engineering of CRISPR/Cas enzymes, based on short synthetic RNAs, has facilitated enormously the introduction of defined genetic and epigenetic modifications in the genome^214^, ^215^ through a variety of approaches including indel‐mediated knockouts, homology‐dependent repair, prime editing, base editing, and epigenetic regulation of transcription. Indeed, recent clinical trials with CRISPR in Transthyretin Amyloidosis,^216^ sickle cell disease and β‐thalassaemia^217^ offer very promising demonstrations of the technology and its exciting potential for patient benefit.

## CONFLICT OF INTEREST STATEMENT

The authors have no conflict of interest.
